# Recombinant *Trichinella pseudospiralis* Serine Protease Inhibitors Alter Macrophage Polarization *In Vitro*

**DOI:** 10.3389/fmicb.2017.01834

**Published:** 2017-09-21

**Authors:** Ning Xu, Xiaolei Liu, Bin Tang, Libo Wang, Hai N. Shi, Pascal Boireau, Mingyuan Liu, Xue Bai

**Affiliations:** ^1^Key Laboratory of Zoonosis Research, Ministry of Education, Institute of Zoonosis, College of Veterinary Medicine, Jilin University Changchun, China; ^2^Yunnan Institute of Parasitic Diseases Puer, China; ^3^Mucosal Immunology Laboratory, Pediatric Gastroenterology Unit, Massachusetts General Hospital, Boston MA, United States; ^4^Laboratory for Animal Health, ANSES, INRA, ENVA, Université Paris-Est Champs-sur-Marne, France; ^5^Jiangsu Co-innovation Center for Prevention and Control of Important Animal Infectious Diseases and Zoonoses Yangzhou, China

**Keywords:** *Trichinella pseudospiralis*, serine proteinase inhibitors, alternatively activated macrophages, inhibitory activity

## Abstract

During parasite infection, serine protease inhibitors secreted by parasites play important roles in suppressing host defenses. However, the mechanism of immune regulation is unclear. In this study, a serpin gene from *Trichinella pseudospiralis*, named *Tp*-Serpin, was cloned and expressed, in order to reveal its role in the regulation of the host immune response in *T. pseudospiralis* infection. The results showed that *Tp*-Serpin encodes a 43 kDa protein that was recognized by serum from *T. pseudospiralis* infected mice at 60 days post-infection (dpi). *Tp*-Serpin was found to be expressed at all developmental stages of *T. pseudospiralis*. Inhibitory activity analysis showed that recombinant *Tp*-Serpin (r*Tp*-Serpin) effectively inhibited the hydrolytic activity of porcine pancreatic elastase (elastase P), human neutrophil elastase (elastase H), and mouse mast cell protease-1, but showed little inhibitory for human neutrophil cathepsin G (cathepsin G). Furthermore, r*Tp*-Serpin induced polarization of macrophages toward the alternatively activated phenotype (M2) alone by activation of the signal transducer and activator of transcription 3 signaling pathway, and inhibited lipopolysaccharide-induced classically activation (M1) *in vitro*. These data preliminarily demonstrate that *Tp*-Serpin may play an important role in the immunoregulation of *T. pseudospiralis* infection by activating the M2-polarized signaling pathway.

## Introduction

*Trichinella* is an intracellular parasite of skeletal muscle that can infect a wide variety of mammalian species and some carnivorous birds. Hosts are infected by ingestion of animal tissues containing infective larvae (Arora et al., 2017). Trichinellosis has been regarded as an emerging or re-emerging widespread food-borne disease (Gottstein et al., 2009; Rostami et al., 2017). *Trichinella*, as with other helminths, can ensure its survival by modulating the host immunological response (Sofronic-Milosavljevic et al., 2015). Induction of host immunosuppression is an important strategy for pathogens to invade their hosts and is a common characteristic of helminth infections. Among the helminths, *Trichinella* is one of the few parasites with the extremely strong ability to induce host immune suppression (Bruschi, 2002). Recently, several studies have shown that *Trichinella* infection can alleviate or inhibit various immune-related diseases, including type I diabetes, experimental allergic encephalitis, inflammatory bowel disease, and airway allergic inflammation (Park et al., 2011; Wang et al., 2016).

Excretory–secretory proteins (ESPs) released by *Trichinella* contain numerous functional proteins, which induce strong immunosuppression in the first 2 weeks of the infection and Th2 polarized and alternatively activated macrophages (M2) respond throughout the whole *Trichinella* infectious process (Ilic et al., 2012). Moreover, the ESPs of *Trichinella* larvae significantly inhibit lipopolysaccharide (LPS)-induced macrophages activity, which play crucial roles in host immune responses against various pathogens (Bai et al., 2012). These studies show that *Trichinella* can regulate the host immune response by encoding immune regulator to interfere with immune recognition. Unfortunately, the key immune regulator of *Trichinella* is still unknown.

Serine protease inhibitors play a variety of important biological roles by controlling endogenous and exogenous proteolytic activities involved in coagulation, inflammation, and apoptosis (Heit et al., 2013). In helminths, serpins play a key role in inhibiting blood coagulation, resisting host protease damage, and also serve as targets for escaping host immune attack (Molehin et al., 2012). These inhibitors also have been shown to play key roles in host immune evasion, and hence the suggestion that helminth serpins may have evolved for the purpose of limiting host immune activation by interfering with host immunomodulatory signals (Molehin et al., 2014). In previous studies, part of a gene encoding serpins from *Trichinella spiralis* and other helminths have been discovered and have shown biological activity (Molehin et al., 2014; Moreira et al., 2014; Zhang et al., 2016). However, the immunomodulatory function of serpins from *Trichinella pseudospiralis* has not yet been reported.

There are nine species and three genotypes in the genus *Trichinella*; some of them develop in muscle cells that become encapsulated (e.g., *T. spiralis*), while others develop in cells and not encapsulated (e.g., *T. pseudospiralis*) (Pozio and Zarlenga, 2013). As it is not encapsulated, *T. pseudospiralis* should be more susceptible to the host immune attack. Relative to *T. spiralis, T. pseudospiralis* induces stronger immunosuppression, to ensure survival in muscle cells (Asano et al., 2016). In the present study, a high-frequency gene encoding a serine protease inhibitor protein from *T. pseudospiralis* (*Tp*-Serpin) was identified. Expression of *Tp*-Serpin has been detected both in ESPs and crude parasite antigen preparations during all development stages of *T. pseudospiralis* suggested that *Tp*-Serpin may play an important role in *T. pseudospiralis* infection. In order to analyze the role of *Tp*-Serpin in regulating the host immune response, recombinant *Tp*-Serpin (r*Tp*-Serpin) was successfully produced in *Escherichia coli* and its function in regulating macrophages polarization was determined.

## Materials and Methods

### Ethics Statement

Animals were treated in strict accordance with the National Institutes of Health guidelines (publication no. 85–23, revised 1996). Studies involving animals were reviewed and approved by the Ethical Committee of Jilin University affiliated to the Provincial Animal Health Committee, Jilin Province, China (Ethical Clearance number IZ-2009-08).

### Cell Culture, Animals, Parasites, and Excretory–Secretory Proteins (ESPs)

BALB/c mice (female, 6–8 weeks old) were purchased from Shanghai SLAC Company. The murine macrophage cell line J774A.1 was purchased from American Type Culture Collection and cultured in RPMI 1640 medium containing 10% heat inactivated fetal bovine serum (FBS) at 37°C in a 5% CO_2_ atmosphere.

*Trichinella pseudospiralis* (ISS13) muscle larvae (ML) were recovered from BALB/c mice at 35 days post-infection (dpi) by pepsin–HCl digestion. Adult worms at day 3 (Ad3) and new born larvae (NBL) were recovered as previously described (Robinson et al., 2007). The ML, Ad3, and NBL were incubated in pre-warmed serum-free RPMI medium 1640 with 2% antibiotics (penicillin and streptomycin) at 37°C and with 5% atmospheric CO_2_ for 24 h. Following incubation the supernatant was collected, dialyzed, and concentrated in using Ultra-15 3K centrifugal filters (Millipore, United states) (Cwiklinski et al., 2009). All parasites and the concentrated ESPs were stored at -80°C for further use.

### Molecular Characterization and Phylogenetic Analysis

The amino acid sequence of *Tp*-Serpin was submitted to https://www.ncbi.nlm.nih.gov/Structure/cdd/wrpsb.cgi to predict its putative structure and reactive-site loop. Phylogenetic relationships among serpins based on *Tp*-Serpin homologs were constructed using MEGA7.

### Cloning, Expression, and Purification of Recombinant *Tp*-Serpin (r*Tp*-Serpin)

Total RNA from ML was collected (Qiagen, Germany) and reverse-transcribed into cDNA (Stratagene, United States). The *Tp-Serpin* sequence (GenBank: JF764789.1) was amplified from the cDNA of ML by PCR (forward primer, 5′-CGC ATA TGC GAT GTC GTC CGT CCG TCA ATT TCG AC-3′, containing the *Nde* I restriction site; reverse primer, 5′-CCG CTC GAG ACC ACG ATA ACT TCC CAT GAA C-3′, containing the *Xho* I restriction site). The PCR products were sub-cloned into pMD19-T-Simple vector (Takara, Dalian, China) for sequencing. The cloned gene was excised by digestion with *Nde* I/*Xho* I and sub-cloned into the pCold I prokaryotic expression vector. After transformation into *E. coli* Rosetta gami (DE3) cells (Novagen, Germany), r*Tp*-Serpin expression was induced with 0.3 mM IPTG for 16 h at 18°C. The r*Tp*-Serpin was purified using HiTrap^TM^ affinity columns (GE healthcare, United States) according to the manufacturer’s instruction. The purified r*Tp*-Serpin was analyzed by 12% SDS–PAGE and Western blot using serum from mice infected with *T. pseudospiralis* for 60 dpi and a HRP-conjugated goat anti-mouse IgG as the secondary antibody (Tang et al., 2015). Western blots were developed using the ECL plus Western blotting detection system (GE Healthcare Buckinghamshire, United Kingdom).

### Production of Polyclonal Antibodies Against r*Tp*-Serpin

Six-week-old BALB/c mice were first injected intraperitoneally with approximately 100 μg of purified r*Tp*-Serpin mixed 1:1 (v/v) with complete Freund’s adjuvant (CFA). Additional injections of 100 μg of protein with incomplete Freund’s adjuvant (IFA) were administered to the animals 2 weeks later. Preimmune serum was collected prior to the immunizations. Two weeks after the last injection, serum samples were collected, titrated, and stored at -20°C.

### Western Blot Analysis of *Tp*-Serpin in *T. pseudospiralis* Developmental Stages

*Trichinella pseudospiralis* worms and the ESPs of NBL, Ad3, and ML, respectively, were prepared as previously described. Equal amounts of samples (20 μg per well, ESPs, and crude protein of NBL, Ad3, and ML) were electrophoresed on 12% SDS–PAGE gel and electro-transferred to PVDF (Immobilon, Millipore, United States). Non-specific binding sites were blocked by immersing the membranes in 5% skim milk in phosphate-buffered saline (PBS) overnight at 4°C. After washing three times in PBS containing 0.1% Tween-20 (PBST), membranes were incubated with mouse anti-r*Tp*-Serpin serum (dilutions 1:200 in PBS) for 1 h at 37°C, and subsequently washed three times in PBST. The membranes were then incubated with HRP-conjugated goat anti-mouse IgG for 1 h at 37°C (diluted 1:5000 in PBS) (Tang et al., 2015). After washing several times with PBST, the peroxidase activity was detected as previously described.

### Inhibitory Activity Assay

Single-stage kinetic assays were used to characterize the inhibitory activity of r*Tp*-Serpin against four serine proteases (Kang et al., 2010). Increasing concentrations of r*Tp*-Serpin (0–15 g/l) were pre-incubated with each of the enzymes in 50 mM PBS (pH 7.4) for 30 min at 25°C followed by the addition of the appropriate chromogenic substrate for 10 min at 25°C. The concentrations (expressed as final concentrations) of enzyme/substrate were shown in **Table 1** (final volume of 200 μl in individual wells of a 96-well microtiter plate). Finally, absorbance changes at 405 nm were monitored over 5 min using a Kinetic Microplate Reader to analyze the hydrolytic activity of the proteases and evaluate the inhibitory activity of recombinant proteins. Reactions without recombinant protein or with phenylmethanesulfonyl (PMSF; Boehringer, Mannheim, Germany) fluoride were employed as negative and positive controls, respectively.

**Table 1:** Enzyme/substrate used for r*Tp*-Serpin inhibitory activity assay.

Enzyme	Substrate
Porcine pancreatic elastase (elastase P, 2 nM, Sigma Aldrich)	*N*-succinyl -Ala-Ala-Ala-*p*-nitroanilide (100 M, Sigma Aldrich)
Human neutrophil elastase (elastase H, 17 nM, Sigma Aldrich)	*N*-succinyl -Ala-Ala-Pro-Leu-*p*-nitroanilide (100 M, Sigma Aldrich)
Human neutrophil cathepsin G (cathepsin G, 220 nM, Sigma Aldrich)	*N*-succinyl -Ala-Ala-Pro-Phe-pnitroanilide (100 M, Sigma Aldrich)
Mouse mast cell protease-1 (mMCP-1, 3 nM, Sigma Aldrich)	*N*-succinyl -Ala-Ala-Pro-Phe-pnitroanilide (100 M, Sigma Aldrich)

### *In Vitro* Treatment of Macrophages

The murine macrophage J774A.1 cells were counted and adjusted to a density of 2 × 10^5^ cells/ml before being cultured in a 96-well cell culture plate (Costar). Cells were stimulated with 1–25 μg/ml r*Tp*-Serpin at 37°C for 48 h in the presence of 5% CO_2_, followed by the addition of 10 μl/well Cell Counting Kit-8 (CCK-8; Dojindo Laboratories, Kumamoto, Japan) solution. Plates were then incubated for 4 h in the dark and the absorbance of the samples at 450 nm was measured.

After the macrophage viability assay, macrophages were treated with r*Tp*-Serpin alone or together with LPS in order to determine the role of r*Tp*-Serpin in macrophage polarization. In the macrophage polarization tests, cells were seeded at a density of 1 × 10^6^ cells/ml and cultured in a 12-well cell culture plates. The cells were treated with r*Tp*-Serpin (5 μg/ml) alone or co-treated with LPS (100 ng/ml) for 24 h. LPS (100 ng/ml) or IL-4 (10 ng/ml) treated cells were used as positive controls. Cell culture medium treated cells were used as a negative control. After 24 h treatment, the cells and conditioned media were collected to be analyzed for polarization activity.

### Flow Cytometry

Treated macrophages were washed in PBS and adjust to 1 × 106 cells/100 μl PBS with 1% FBS. Cells were incubated with antibodies against CD206 (PE-conjugated, 0.5 μg per million cells in 100 μl volume) and CD16/32 (FITC-conjugated, 1.0 μg per million cells in 100 μl volume) (BioLegend, United States) for surface marker analysis of the polarized macrophages. Cell suspensions were incubated with the antibodies for 30 min at 4°C and washed three times with PBS. Samples were analyzed using a BD FACSCalibur Flow Cytometer and the results were analyzed using FlowJo software (Tree star Inc., Ashland, OR, United States).

### Real-Time PCR

Treated macrophages were washed with RPMI 1640 medium. RNA was extracted and purified (Qiagen, Germany) and converted to cDNA (Stratagene, United States) according to the manufacturer’s instructions. Quantitative real-time PCR was conducted using FastStart Universal SYBR Green Master (Rox) reagents (Roche Diagnostics, Indianapolis, IN, United States) and a 7500 real-time PCR machine (Applied Biosystems, Foster City, CA, United States). The reaction conditions were: 1, 95°C for 10 min; 2, 40 cycles of 95°C for 15 s, 56°C for 1 min, and 72°C for 1 min, which were concluded by a melting curve analysis. Fold changes of gene expression were calculated using the 2^-ΔΔC_T_^ method. Primer sequences are listed in **Table 2** (Bai et al., 2012).

**Table 2:** Primers used for quantitative real-time PCR.

Gene	Primer sequences (5′→3′)	Accession number	Length (bp)
GAPDH	Forward: CTGCCCAGAACATCATCCCT Reverse: GGTCCTCAGTGTAGCCCAAGA	NM_008084	234
IL-1β	Forward: CCTCGTGCTGTCGGACCCATA Reverse: CAGGCTTGTGCTCTGCTTGTGA	NC_000068.6	344
IL-10	Forward: CCTCAGTTCCCATTCTATTTATTCACT Reverse: TTGAAAGGACACCATAGCAAAGG	NC_000067.5	255
IL-12	Forward: TGACACCTTTGCTGATTTCTAC Reverse: TCTCCAAATACCACCTATGTCTT	M86671	375
IFN-γ	Forward: GTGGCATAGATGTGGAAGAAA Reverse: TGCTGATGGCCTGATTGTC	NM_008337	147
TGF-β	Forward: GAGGCGGTGCTCGCTTTGTA Reverse: CTTCCCGAATGTCTGACGTATTG	NC_000073.5	205
iNOS	Forward: ACATTCAGATCCCGAAACGC Reverse: GACAATCCACAACTCGCTCC	NC_000076.5	312
Arg1	Forward: GGGGAAAGCCAATGAAG Reverse: TGGTTGTCAGGGGAGTGT	NM_010927.3	212

### Cytokine Assays

To assay cytokine production levels, supernatants from treated macrophages were collected. Levels of pro-inflammatory (IFN-γ, IL-1β) and anti-inflammatory cytokines (IL-10, TGF-β) production were analyzed using the murine cytokine ELISA Kit (eBioscience, United States). Cytokine concentrations were determined in duplicate and at a dilution that fell in the middle of the standard curve according to the manufacturer’s protocol. All measurements were performed in triplicate. The average absorbance at 450 nm was determined for each sample and was used to calculate cytokine concentrations as picograms per milliliter (pg/ml).

### Analysis of STAT3/JAK2 Phosphorylation and the Relationship with IL-10 Levels

In the signaling pathway analysis, J774A.1 macrophages (1 × 10^6^ cells/well) were cultured in 6-well plates for 24 h and were treated with different concentrations of r*Tp*-Serpin (0.5, 1, and 2 μg/ml) or cell culture medium for 2 h. The conditioned medium was collected to assay IL-10 levels by ELISA as described previously. Cells were then collected, washed three times with ice-cold PBS, and re-suspended in PBS mixed with 1 mM PMSF (Boehringer, Mannheim, Germany). After incubation on ice for 30 min, protein concentrations were measured with the Pierce BCA Protein Assay Kit (Illinois, United States). For the analysis of signal transducer and activator of transcription 3 (STAT3)/JAK2 phosphorylation, 30 μg of total cellular protein was analyzed by Western blot with rabbit anti-mouse STAT3/JAK2 antibody, rabbit anti-mouse p-STAT3/JAK2 antibody (Santa, CA, United States), and β-actin (mouse monoclonal Ab6276), followed by a HRP-conjugated goat anti-rabbit secondary antibody (1:20,000). STAT3/JAK2 activation was detected as previously described.

### Statistical Analysis

All results were expressed as mean ± SD. Statistical analysis was performed using the GraphPad Prism 5 for Windows. One-way and two-way analysis of variance (ANOVA) was used to compare statistical differences at different conditions. *P*-values are expressed as ^∗^*p* < 0.05 and ^∗∗^*p* < 0.01 in comparison with the control group or LPS treatment group.

## Results

### Molecular Characterization of the *Tp*-Serpin

**FIGURE 1:**
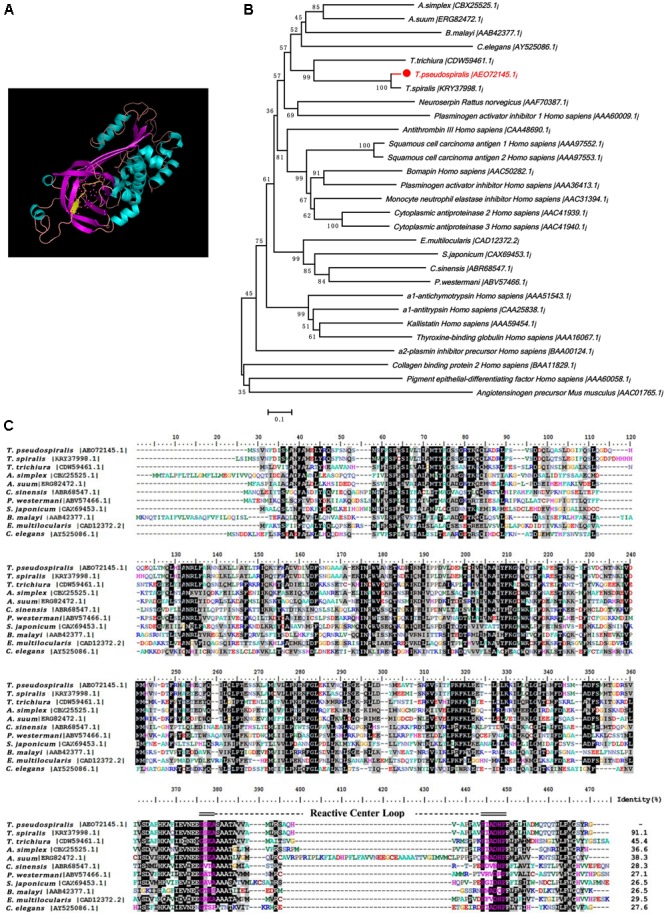
Putative structures and the phylogenetic relationship of *Tp*-Serpin to other serpin homologs. **(A)** Structure of *Tp*-Serpin. The reactive-site loop of the serpins is shown as a yellow strand. **(B)** Phylogenetic tree of *Tp*-Serpin and other serine proteinase inhibitors. **(C)** Amino acid sequences of *Tp*-Serpin in the region of the reactive-site loop and comparison with other nematode serpin proteins. The reactive-site loop is shaded in blue.

The *Tp*-Serpin open-reading frame was found to 1134 bp and encoded 378 amino acids. Based on the structural prediction, *Tp*-Serpin consists of 60.71% α-helices and 39.29% β-sheets, and the solvent-exposed reactive center loop is near the C-terminus (**Figures 1A,C**). The phylogenetic relationship of *Tp*-Serpin with serpin family proteins was analyzed by comparing amino acid sequences. The results indicated that *Tp*-Serpin is genetically related to *Ts*-Serpin, from *T. spiralis*, and are more closely related to nematode serpins than vertebrate serpins (**Figure 1B**).

### Cloning, Expression, and Purification of Recombinant *Tp*-Serpin (r*Tp*-Serpin)

**FIGURE 2:**
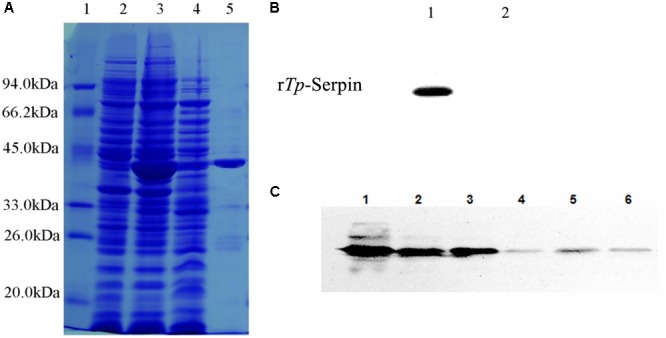
Analysis of purified recombination *Tp*-Serpin (r*Tp*-Serpin) and the expression pattern of *Tp*-Serpin in *T. pseudospiralis* developmental stages. **(A)** SDS–PAGE of purified r*Tp*-Serpin M: protein standard; 1: total protein expressed without induction; 2: total protein induced by 0.3 mM IPTG; 3: soluble protein induced by 0.3 mM IPTG; and 4: purified rTp-Serpin. **(B)** Antigenicity of r*Tp*-Serpin as determined by Western blot analysis. 1: sera of mice at 60 dpi and 2: preimmune sera of mice. **(C)** Western bolt analysis of *Tp*-Serpin in *T. pseudospiralis* developmental stages. 1: *T. pseudospiralis*-AD3 crude parasite antigen, 2: *T. pseudospiralis*-NBL crude parasite antigen, 3: *T. pseudospiralis*-ML crude parasite antigen, 4: *T. pseudospiralis*-AD3 ESPs, 5: *T. pseudospiralis*-NBL ESPs, and 6: *T. pseudospiralis*-ML ESPs.

The full-length *Tp*-Serpin gene was obtained, cloned into the prokaryotic expression vector pCold I, and transformed into *E. coli* Rosetta gami (DE3). The recombinant protein (r*Tp*-Serpin) was expressed in *E. coli* as a soluble protein with a relative molecular mass of about 43k Da (**Figure 2A**), which was consistent with the estimated molecular mass of the deduced amino acid sequence of *Tp*-Serpin. The purified r*Tp*-Serpin was recognized specifically by serum from mice infected with *T. pseudospiralis* at 60 dpi (**Figure 2B**).

### *Tp*-Serpin Expression in All Life Stages of *T. pseudospiralis*

*Tp*-Serpin expression was detected throughout all examined developmental stages, including ML, Ad3, and NBL (**Figure 2C**). To investigate the expression pattern of *Tp*-Serpin, the ESPs and crude parasite antigens from *T. pseudospiralis* at all developmental stages were analyzed by Western blotting. Anti-r*Tp*-Serpin serum recognized an abundant amount of *Tp*-Serpin in a band at approximately 43 kDa in crude parasite antigen, and ESPs also showed a weak positive reaction (**Figure 2C**). This suggests that *Tp*-Serpin was expressed at all developmental stages of *T. pseudospiralis*, and was a secretory protein, which play an important role in regulate the host immune response.

### Inhibitory Activity of r*Tp*-Serpin *In Vitro*

**FIGURE 3:**
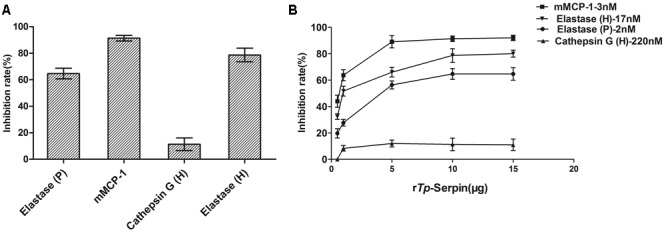
Inhibitory activity assay of r*Tp*-Serpin against different proteases. **(A)** Inhibitory activity assay of r*Tp*-Serpin on porcine pancreatic elastase (elastase P), human neutrophil elastase (elastase H), human neutrophil cathepsin G (cathepsin G), and mouse mast cell protease-1 (mMCP-1). **(B)** Concentration-dependent inhibition profiles of elastase P, elastase H, cathepsin G, and mMCP-1.

To study the potential inhibitory activity of r*Tp*-Serpin, inhibition of a series of serine proteases with different substrate specificity was tested. Inhibitory activity assays showed that r*Tp*-Serpin effectively inhibited the hydrolysis activity of elastase (P/H) and mouse mast cell protease-1 (mMCP-1), but showed little inhibitory activity against cathepsin G (**Figure 3A**). In addition, the inhibitory activity of r*Tp*-Serpin appeared to be dose dependent (**Figure 3B**).

### The Viability of Macrophages Treated with r*Tp*-Serpin *In Vitro*

**FIGURE 4:**
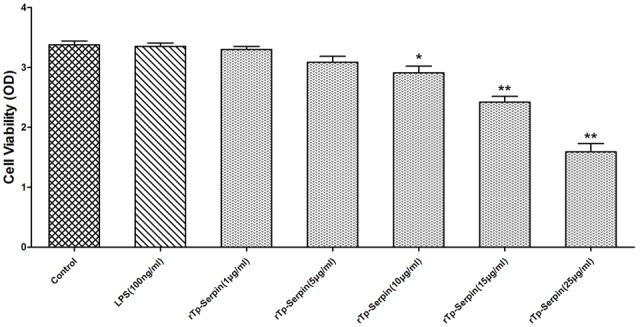
The viability of J774A.1 macrophages treated with r*Tp*-Serpin. Effect of different concentration r*Tp*-Serpin (1–25 μg/ml) on the viability of J774A.1 macrophages. Results are shown as the mean ± standard deviation (SD) for at least five independent experiments for each group. Significant differences were as follows: ^∗^*p* < 0.05; ^∗∗^*p* < 0.01.

To further investigate the function of r*Tp*-Serpin in the polarization of J774A.1 macrophages, a CCK-8 assay was performed. As shown in **Figure 4**, at low concentrations (1–5 μg/ml), r*Tp*-Serpin did not affect the viability of macrophages, while it had a significant difference in high concentration (*p* < 0.05, 10 μg/ml; *p* < 0.01, 15–25 μg/ml). In view of the above results, the concentration of recombinant protein required for macrophage differentiation was 5 μg/ml.

### Phenotype Analysis of Macrophage by r*Tp*-Serpin

**FIGURE 5:**
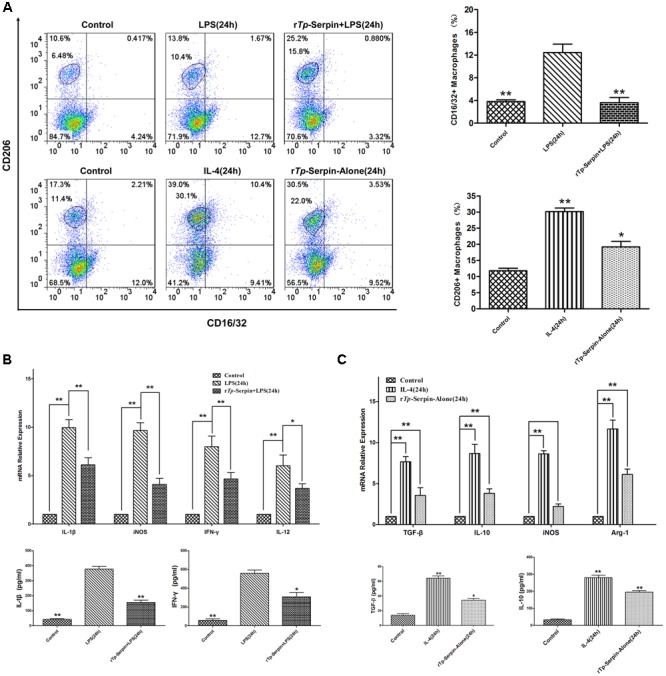
The polarization J774A.1 macrophages treated with r*Tp*-Serpin. **(A)** Flow cytometry analysis macrophage polarization and expression of CD16/32 (M1) and CD206 (M2) after treatment with r*Tp*-Serpin. **(B)** Effect of rTp-Serpin on pro-inflammatory cytokines and iNOS expression in LPS-induced J774A.1 macrophages. J774A.1 macrophage cells were treated with rTp-Serpin (5 μg/ml) + LPS (100 ng/ml) or LPS (100 ng/ml) alone for 24 h. **(C)** Effect of rTp-Serpin on anti-inflammatory cytokines and marker effector molecules (iNOS, Arg1) in J774A.1 macrophages. J774A.1 macrophage cells were treated with 5 μg/ml rTp-Serpin alone or IL-4 (10 ng/ml) for 24 h. Control group was no treatment/medium alone. Results are shown as the mean ± SD for at least three independent experiments for each group. Significant differences are as follows: ^∗^*p* < 0.05; ^∗∗^*p* < 0.01.

In flow cytometry analysis, the percentage of CD206^+^ macrophage cells was found to be significantly increased after incubation with r*Tp*-Serpin alone compared with the control group (*p* < 0.05, **Figure 5A**). On the other hand, r*Tp*-Serpin significantly suppressed the percentage of CD16/32^+^ macrophages induced by LPS compared with the LPS group (*p* < 0.01, **Figure 5A**).

In SYBR green I real-time PCR analysis, the mRNA levels of pro-inflammatory cytokines (IFN-γ, IL-1β, and IL-12) were suppressed by r*Tp*-Serpin in LPS-treated macrophages compared with the LPS group (*p* < 0.05, IL-12; *p* < 0.01, IFN-γ and IL-1β; **Figure 5B**). Additionally, the level of iNOS was inhibited in a similar fashion to the pro-inflammatory cytokines (**Figure 5B**). Furthermore, in the tests of macrophages treated with r*Tp*-Serpin alone, the expression of anti-inflammatory cytokines (IL-10, TGF-β) and marker effector molecules of M2 (Arg1) was significantly up-regulated (*p* < 0.01, **Figure 5C**). Moreover, the level of iNOS showed no significant change following stimulation with r*Tp*-Serpin (**Figure 5C**). Similarly, in the ELISA test, the expression levels of pro-inflammatory (IFN-γ, IL-1β) and anti-inflammatory cytokines (IL-10, TGF-β) demonstrated the same biological effect (*p* < 0.01, IL-1β and IL-10; *p* < 0.05, IFN-γ and TGF-β; **Figures 5B,C**). In summary, r*Tp*-Serpin induced polarization of macrophages toward the M2 phenotype, and inhibited LPS-induced M1 polarization *in vitro*.

### r*Tp*-Serpin Activates the JAK2/STAT3 Signaling Pathway

**FIGURE 6:**
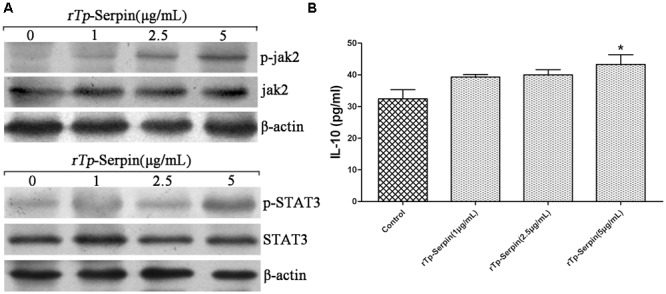
Analysis of phosphorylation in JAK2/STAT3 signaling pathway and the expression levels of IL-10 in the early period of M2 polarization after treatment with r*Tp*-Serpin. **(A)** Western bolt analysis of phosphorylation in JAK2 and STAT3 after treatment with different concentrations of r*Tp*-Serpin (1–5 μg/ml). **(B)** Expression levels of IL-10 in the early period of M2 polarization. Results are shown as the mean ± SD for at least three repeated experiments for each group. Significant differences are as follows: ^∗^*p* < 0.05; ^∗∗^*p* < 0.01.

To determine whether the polarization of macrophages treated by r*Tp*-Serpin was secondary to the activation of a specific upstream signaling pathway within the macrophage, phosphorylation of JAK2/STAT3 was evaluated to determine the effect on the activation state of macrophages. Western blot analysis demonstrated a striking phenotypic difference between macrophages treated with different concentrations of r*Tp*-Serpin. The phosphorylation of STAT3 and JAK2 increased with increasing doses of r*Tp*-Serpin (**Figure 6A**). Unexpectedly, IL-10 levels were not significantly different to the negative control group in low concentration of r*Tp*-serpin (1–2.5 μg/ml, **Figure 6B**). This indicated that phosphorylation was detected before IL-10 up-regulation.

## Discussion

Parasite serpins play an important role in interference with immune recognition and the immune response in the process of invasion in the body. Studies have shown that the *Schistosoma haematobium* serpin reduced immunogenicity of the pathogen by binding with human trypsin and evading the host’s immune attack (Molehin et al., 2012). In addition, some parasite serine protease inhibitors are involved in embryonic development and reproductive processes, mediated by endogenous modulators that act on the protease (Nagano et al., 2001). In our study, the putative structure of *Tp*-Serpin was shown to have a common highly ordered tertiary structure that is shared with all members of the serpin family. The functional domain, known as reactive-site loop, is near the C-terminus and is exposed on the surface of the protein, which traps the protease. In summary, based on a detailed comparison between amino acid sequences of *Tp*-Serpin and other members of the serpin family, it was concluded that *Tp*-Serpin has the inhibition activity, which was more closely related to other serpins.

Generally, serpins of parasitic helminthes have strong immunoreactivity and are classified into secretory and intracellular categories (Molehin et al., 2012). In our study, the results showed that the r*Tp*-Serpin was specifically recognized by serum from mice infected with *T. pseudospiralis* for 60 days. *Tp*-Serpin induced the humoral immune response in mice and may act as the main protective antigen in the infection process of *T. pseudospiralis*. Analysis of various life-cycle stages of *T. pseudospiralis* for the expression of *Tp*-Serpin showed that translation of *Tp*-Serpin happened in all periods of *T. pseudospiralis* development, suggested that *Tp*-Serpin may play an important role in the development of *T. pseudospiralis.* Furthermore, *Tp*-Serpin was detected in excretory–secretory proteins (ESPs), indicated that it is an exocrine protein and may function by acting directly on cells or humoral molecules of the host.

*Trichinella* have the ability to evade the host immune response, which results in forming a long-term infection in the host. At present, two clades of *Trichinella* have been identified: encapsulated species (*T. spiralis*) and non-encapsulated species (*T. pseudospiralis*) (Bruschi et al., 2014). Both of these clades exert strong immunosuppression in order to evade the host immune response. However, as *T. pseudospiralis* is non-encapsulated it should be more susceptible to host immune attack. Therefore, *T. pseudospiralis* must produce stronger immunosuppression to live in muscle cells. In both primates and rodents, *T. pseudospiralis* is less pathogenic than *T. spiralis*, generating considerably less inflammation (due to strong immunosuppression) (Reichard et al., 2015). These differences also extend to the suppression of cellular infiltration and diffuse myopathy (Boonmars et al., 2005). So far, the studies related to the genes involved in *T. pseudospiralis* immune escape are undefined. However, research on the immune evasion of serine protease inhibitors (Serpins) during the invasion period of other helminths provides some indications (Nagano et al., 2003). In this current study, the hydrolytic activity of trypsin, elastase, and chymotrypsin was significantly inhibited by r*Tp*-Serpin. This suggests that *Tp*-Serpin may have biological activity and play important function in evasion host immune response in each stage of *T. pseudospiralis* natural infection by inhibiting the function of enzymes secreted host cell. As elastase (P) and mMCP-1 are located in the digestive tract, the inhibition of digestion enzymes may prevent *T. pseudospiralis*, in the intestinal infection stage, from being damaged. Moreover, mMCP-1 plays a role in chemotaxis of neutrophils and other inflammatory cells and enhances the permeability of intestinal epithelial cells, thereby enabling complement and antibodies into the intestine to kill and exclude parasites (Miller and Pemberton, 2002). Therefore, interaction with r*Tp*-Serpin can offset the immune response and affect the exclusion caused by mMCP-1 and elastase (P) in the intestinal tract. Unlike elastase (P) and mMCP-1, elastase (H) can promote the production of inflammatory cytokines and antibodies, as well as lymphocyte proliferation (Bank and Ansorge, 2001). These positive effects of immune response also help to prevent parasite invasion in the host (Molehin et al., 2012). Thus, by inhibiting the biological activity of elastase (H), r*Tp*-Serpin may contribute *T. pseudospiralis* ability to evade the body’s immune response.

Generally, in response to environmental stimuli, the balance of the Th1/Th2 immune response changes, and macrophage polarization can take place in the appropriate immune response environment, leading to M1 or M2 phenotypes (Gordon, 2003; Biswas and Mantovani, 2010; Sudduth et al., 2013). It is well accepted that parasites can strongly induce M2 polarization (Ramanan et al., 2016; Kim et al., 2017). During the process of M2 polarization, the types of cytokines and effector molecules in macrophages are changed, and leading to anti-inflammatory and tissue repair effects (Kreider et al., 2007; Lumeng et al., 2007; Killock, 2011; Lim et al., 2016; Bosurgi et al., 2017). Similarly, previous studies have shown that Arg1 can inhibit the pro-inflammatory function of iNOS. In our study, we tested the ability of r*Tp*-Serpin to induce macrophage polarization by incubation with murine J774A.1 macrophages alone or co-treatment with LPS. As expected, M2 polarization and inhibition of LPS-induced M1 polarization were confirmed by the percentage changes in CD206^+^ (M2) or CD16/32^+^ (M1) cells, respectively. Furthermore, the transcription and expression of anti-inflammatory cytokines and functional molecules of M2 (Arg1) were up-regulated by r*Tp*-Serpin alone. On the other hand, pro-inflammatory cytokines and iNOS induced by LPS were suppressed, indicating that r*Tp*-Serpin inhibited macrophage polarization to the M1 phenotype by co-treatment with LPS. Taken together, these results suggest that r*Tp*-Serpin alone could induce M2 polarization. Similarly, r*Tp*-Serpin inhibited M1 polarization caused by LPS *in vitro*.

In the process of M2 polarization and helminth infection, the immune response of the host to helminth infections is strikingly dominated by a Th2 response with a significant production of IL-4, IL-10, IL-13 (Pulendran and Artis, 2012), and the STAT3/STAT6 signaling pathways have been detected (O’Shea et al., 2002; Schindler et al., 2007). Unlike *T. spiralis* and most other helminths, *T. pseudospiralis* induces strong immunosuppression and inhibits both Th1 and Th2 immune responses (Dvoroznakova et al., 2011), in which the IL-4/STAT6 signaling pathway does not have such an effect. Simultaneously, high levels of IL-10 were also detected locally (Bastos et al., 2002; Beiting et al., 2007). Consistent with previous results, all pro-inflammatory cytokines levels in macrophages treated with LPS, including IL-1β (Ip et al., 2017), were suppressed by r*Tp*-Serpin, which implies that the alternation of macrophage polarization induced by r*Tp*-Serpin may be IL-4/STAT6-independent *in vitro*. Unlike other Th2 cytokines, the IL-10 signaling pathway recruits Jak2 followed by phosphorylation of tyrosine in STAT3, and induces the expression of the suppressor of cytokine signaling (SOCS3), which can reduce the expression of a variety of cytokines (Nakamura et al., 2015). Combined with the inhibition of macrophage pro-inflammatory cytokines, STAT3 was found to be responsible for the effect of pro-inflammatory cytokine suppression by Western blot analysis. Since phosphorylation of STAT3 was detected earlier than an increase in IL-10, r*Tp*-Serpin may activate other receptors to phosphorylate STAT3 in an IL-10R-independent method *in vitro*, which needs to be confirmed in further study.

## Conclusion

We have determined that r*Tp*-Serpin could effectively inhibit the immune response by inhibiting the hydrolytic activity of immune-related proteases *in vitro*. M2 polarization was confirmed by flow cytometry and the up-regulation of M2-associated genes (cytokines and effector molecules) through STAT3 signaling pathway activation was also confirmed. Moreover, *Tp*-Serpin inhibited LPS-induced M1 polarization by inhibition of pro-inflammatory cytokines. Although the immunoregulatory activity requires further verification *in vivo*, it appears that r*Tp*-Serpin may play an important role in the regulation of *T. pseudospiralis* infection by macrophage polarization. Furthermore, we believe that *Tp*-Serpin may have the potential to reduce damage in autoimmune diseases.

## Author Contributions

NX analyzed data and wrote the paper; XB and XL designed the research; XL, BT, LW, HS, and PB performed the experiments; ML approved the version to be published; and all authors read and approved the final manuscript.

## Conflict of Interest Statement

The authors declare that the research was conducted in the absence of any commercial or financial relationships that could be construed as a potential conflict of interest.
